# A Challenge for the Development of Malaria Vaccines: Polymorphic Target Antigens

**DOI:** 10.1371/journal.pmed.0040116

**Published:** 2007-03-13

**Authors:** Colin Sutherland

Parasites of the genus Plasmodium cause many hundreds of millions of cases of malaria worldwide optimism that in the future effective vaccination will join the current strategies of preventive and therapeutic uses of antimalarials, and of reduction in human–vector contact, as part of the global malaria control toolkit.

## Malaria vaccine targets

The complex life cycle of the malaria parasite, a protozoan of the phylum Apicomplexa, requires a sophisticated array of proteins. These are encoded by a genome of 23 Mb distributed across 14 chromosomes in P. falciparum [1], significantly larger than the genome of any human pathogen for which effective vaccines have been successfully developed. Vaccine candidates for P. falciparum and P. vivax that have advanced to clinical trials in recent years are targeted against two distinct stages of the parasite life cycle. The first is the sporozoite, which is injected by the bite of a mosquito into the human host as a haploid, free-living unicellular form, and which seeks out the liver, where it invades a hepatocyte and undergoes intracellular multiplication. Among key target antigens at this stage are thrombospondin-related adhesive protein (TRAP), liver-stage antigen 1 (LSA-1) and circumsporozoite protein (CSP). The most successful malaria vaccine to date, the recombinant protein RTS,S administered with the adjuvant AS02A, afforded sustained protection to ~30% of children under five years of age in a large proof-of-principle Phase II trial in Mozambique [2]. This vaccine is based on the CSP antigen, and is designed to prevent infection.

**Glossary**

**CSP:** Circumsporozoite protein, a candidate sporozoite vaccine antigen; primary component of the RTS,S vaccine

**Haplotype:** Literally a “haploid genotype.” As used in this article, refers to a particular combination of linked polymorphisms occurring in a particular parasite clone

**LSA-1:** Liver-stage antigen 1; candidate liver-stage vaccine antigen

**Merozoite:** Free-living parasite that emerges from infected hepatocytes and invades erythrocytes to initiate disease-causing blood-stage malaria; replication in erythrocytes releases further merozoites every 48–72 hours

**MSP-1:** Merozoite surface protein 1; a large multi-domain protein associated with the merozoite apical complex; cleaved by specific proteases during erythrocyte invasion

**MSP-1_19_:** Carboxyl-terminal domain of MSP-1 retained on the merozoite surface after proteolytic cleavage; quite conserved in sequence, and shown to elicit protective immune responses in a number of studies; a leading candidate blood-stage antigen

**Sporozoite:** Free-living malaria parasite stage injected into vertebrate host by mosquito bite; invades hepatocytes

**TRAP:** Thrombospondin-related adhesive protein, a candidate sporozoite/liver stage vaccine antigen

A second major class of malaria vaccines targets the blood stage of the life cycle. This intraerythrocytic stage of infection is responsible for the syndrome of clinical symptoms familiar to us as malaria. Free-living merozoites in the blood, before invading a host erythrocyte, present the immune system with a number of potential immunogens. Among these, merozoite surface protein 1 (MSP-1) is considered one of the most promising vaccine targets, and a number of candidate MSP-1 vaccines are currently in the development pipeline. Many of these are based on the 19 kDa polypeptide at the carboxyl terminus of the MSP-1 protein, MSP-1_19_. As a prime target of natural immune responses in malaria-exposed populations, MSP-1 is a polymorphic antigen, and many variants can occur in a single parasite population. Therefore, there is a risk that MSP-1 vaccine-elicited immune responses may be variant specific, and thus not provide protection against all parasite genotypes encountered within a given population. The MSP-1_19_ portion of the molecule is relatively conserved, but does contain six polymorphic amino acid residues that may contribute to immune evasion by the parasite.

## Developing the Capacity for Malaria Vaccine Trials—Bandiagara, Mali

Renewed interest in the testing of malaria vaccines at a number of clinical trial sites in sub-Saharan Africa has lead to development of the infrastructure and expertise required for Phase II and Phase III studies of vaccine safety, immunogenicity, and efficacy. One of these sites is at Bandiagara, Mali, where malaria transmission is intense but highly seasonal. In this month's *PLoS Medicine*, Shannon Takala and colleagues present a detailed longitudinal analysis of polymorphisms in the *msp-1* genes of parasite isolates taken from a cohort of a broad age range in Bandiagara [3]. Monthly peripheral blood samples (*n* = 2,309) from a random selection of 100 cohort participants across three age strata were analysed through three malaria transmission seasons from 1999 to 2001. This group of 100 individuals provided a staggering 1,375 parasite-positive events during this time, and these isolates were analysed for *msp-1* polymorphisms by pyrosequencing. The authors use the data to present an analysis of the population-level dynamics of 14 different haplotypes encoding MSP-1_19_. The study provides novel and important information on three levels.

First, Takkala and colleagues are able to measure the prevalence of haplotypes in the different MSP-1_19_ population and demonstrate dynamic fluctuations over the three-year study. These data can inform vaccine design, by indicating that either of two dominant haplotypes (QKSNGL and EKSNGL, respectively, at the six polymorphic amino acid positions) occur in about 80% of infections overall. In contrast, the haplotype ETSSRL, found in the 3D7 laboratory clone of P. falciparum, and the basis of one leading MSP-1 vaccine, FMP1/AS02A, occurred in only 16% of infections. Thus, if vaccine-elicited immunity is sequence anti-MSP-1_19_ specific, a vaccine targeting either or both of the more prevalent haplotypes might be more effective in this population. Interestingly, the 3D7 haplotype ETSSRL, and two other haplotypes, appeared to be significantly more common in asymptomatic infections, a result largely accounted for in multivariate modelling by an association with lower parasite densities. This raises the intriguing possibility that specific MSP-1_19_ haplotypes may contribute to reduced virulence, but as Takkala et al. point out, this possibility requires further investigation in other endemic areas, and with a broader genome-wide analysis of parasite polymorphism.

Second, it was found that the seasonality of malaria transmission in Mali has a profound effect on the prevalence of some MSP-1_19_ haplotypes, on the average number of parasite clones in each infection, and on the relative proportion of parasite-positive individuals who had symptomatic malaria, as opposed to asymptomatic infection, in each age group. Such “baseline” knowledge of parasite population dynamics, collected in the absence of a vaccine intervention, is crucial to any future analysis of postintervention data.

Third, careful analyses of sequential isolates collected from individual patients provide new evidence that some MSP-1_19_ haplotypes elicit sequence-specific immunity. For a given surveillance interval in which two infections occurred in the same patient, infection with a haplotype differing in amino acid sequence at residue 1691, 1700, or 1716 was significantly associated with risk of clinical malaria in the latter episode. This finding suggests that there is some level of haplotype-specific immunity, and may also mean that only a subset of the possible MSP-1_19_ sequence combinations needs to be covered by a vaccine in order to counteract the effect of polymorphism at these residues.

## Gathering Intelligence

The data presented by Takkala et al., gathered in the absence of any intervention with a vaccine, demonstrate the potential impact that parasite population diversity could have on the outcome of MSP-1_19_ vaccine trials. Confirmation in other endemic settings is required to verify the evidence that certain residues in this antigen may be particularly important in eliciting sequence-specific protection, and that particular haplotypes are associated with lower parasite densities. Nevertheless, this study provides ample warning that analysis of antigen diversity in the target parasite population should not only be gathered as part of postintervention evaluation in vaccine trials [4], but should be part of the intelligence gathering undertaken when planning intervention studies in the first place.
