# Correction: A Challenge for the Development of Malaria Vaccines: Polymorphic Target Antigens

**DOI:** 10.1371/journal.pmed.0040175

**Published:** 2007-05-29

**Authors:** Colin Sutherland

In *PLoS Medicine*, volume 4, issue 3, doi: 10.1371/journal.pmed.0040116:

In this Perspective, the surname of the lead author of the related research article [Takala SL, Coulibaly D, Thera MA, Dicko A, Smith DL, et al. (2007) Dynamics of polymorphism in a malaria vaccine antigen at a vaccine-testing site in Mali. PLoS Med 4: e93. doi:10.1371/journal.pmed.0040093] was misspelled as Takkala in all but the first use. The correct spelling is Takala.

In addition, the amino acid residues cited in the following sentence should be “1691, 1701, or 1716,” rather than “1691, 1700, or 1716”:

“For a given surveillance interval in which two infections occurred in the same patient, infection with a haplotype differing in amino acid sequence at residue 1691, 1700, or 1716 was significantly associated with risk of clinical malaria in the latter episode.”

